# Inhibition of Bone Morphogenetic Protein Signal Transduction Prevents the Medial Vascular Calcification Associated with Matrix Gla Protein Deficiency

**DOI:** 10.1371/journal.pone.0117098

**Published:** 2015-01-20

**Authors:** Rajeev Malhotra, Megan F. Burke, Trejeeve Martyn, Hannah R. Shakartzi, Timothy E. Thayer, Caitlin O’Rourke, Pingcheng Li, Matthias Derwall, Ester Spagnolli, Starsha A. Kolodziej, Konrad Hoeft, Claire Mayeur, Pawina Jiramongkolchai, Ravindra Kumar, Emmanuel S. Buys, Paul B. Yu, Kenneth D. Bloch, Donald B. Bloch

**Affiliations:** 1 Cardiovascular Research Center and Cardiology Division of the Department of Medicine, Massachusetts General Hospital, Harvard Medical School, Boston, MA, United States of America; 2 Anesthesia Center for Critical Care Research of the Department of Anesthesia, Critical Care, and Pain Medicine, Massachusetts General Hospital, Harvard Medical School, Boston, MA, United States of America; 3 Department of Anesthesiology, Uniklinik Aachen, RWTH Aachen University, Aachen, Germany; 4 Acceleron Pharma, Inc. Cambridge, MA, United States of America; 5 Cardiovascular Division, Brigham and Women’s Hospital, Harvard Medical School, Boston, MA, United States of America; 6 Center for Immunology and Inflammatory Diseases and the Division of Rheumatology, Allergy, and Immunology of the Department of Medicine, Massachusetts General Hospital, Harvard Medical School, Boston, MA, United States of America; Brigham and Women’s Hospital, Harvard Medical School, UNITED STATES

## Abstract

**Objective:**

Matrix Gla protein (MGP) is reported to inhibit bone morphogenetic protein (BMP) signal transduction. MGP deficiency is associated with medial calcification of the arterial wall, in a process that involves both osteogenic transdifferentiation of vascular smooth muscle cells (VSMCs) and mesenchymal transition of endothelial cells (EndMT). In this study, we investigated the contribution of BMP signal transduction to the medial calcification that develops in MGP-deficient mice.

**Approach and Results:**

MGP-deficient mice (MGP^-/-^) were treated with one of two BMP signaling inhibitors, LDN-193189 or ALK3-Fc, beginning one day after birth. Aortic calcification was assessed in 28-day-old mice by measuring the uptake of a fluorescent bisphosphonate probe and by staining tissue sections with Alizarin red. Aortic calcification was 80% less in MGP^-/-^ mice treated with LDN-193189 or ALK3-Fc compared with vehicle-treated control animals (P<0.001 for both). LDN-193189-treated MGP^-/-^ mice survived longer than vehicle-treated MGP^-/-^ mice. Levels of phosphorylated Smad1/5 and Id1 mRNA (markers of BMP signaling) did not differ in the aortas from MGP^-/-^ and wild-type mice. Markers of EndMT and osteogenesis were increased in MGP^-/-^ aortas, an effect that was prevented by LDN-193189. Calcification of isolated VSMCs was also inhibited by LDN-193189.

**Conclusions:**

Inhibition of BMP signaling leads to reduced vascular calcification and improved survival in MGP^-/-^ mice. The EndMT and osteogenic transdifferentiation associated with MGP deficiency is dependent upon BMP signaling. These results suggest that BMP signal transduction has critical roles in the development of vascular calcification in MGP-deficient mice.

## Introduction

Calcification of the intimal and medial layers of the arterial wall is an important risk factor for cardiovascular events [1, 2, 3, 4]. Intimal and medial calcification are the results of different underlying pathogenic mechanisms [5, 6]. Intimal calcification is preceded by subintimal lipid deposition and macrophage accumulation whereas medial calcification is not associated with lipid deposition or inflammation and results from metabolite-induced upregulation of osteogenic gene programs in the vasculature [5, 6]. The processes of intimal and medial vascular calcification have been likened to bone formation, of which there are two types: Intimal atherosclerotic calcification displays similarities to endochondral ossification, involving chondrogenesis prior to bone formation; medial vascular calcification is similar to intramembranous bone formation in which bone derives from mesenchymal stem cells that have differentiated directly into osteoblasts [7, 8].

Matrix Gla protein (MGP) is an extracellular polypeptide that inhibits arterial calcification [9]. Mutations in the *MGP* gene are associated with Keutel syndrome [10], a rare autosomal recessive disease characterized by calcification of the coronary, cerebral, hepatic, and renal arterial beds [10, 11, 12, 13]. Common sequence variants in the *MGP* gene are associated with increased risk and progression of coronary calcification in humans [14, 15]. MGP requires γ-carboxylation of glutamic acid residues for activity, a process that depends on vitamin K as a cofactor and is inhibited by warfarin [9, 16]. Mice lacking both copies of the *mgp* gene spontaneously develop medial arterial calcification beginning at 2 weeks of age. Vascular calcification progresses over time and results in aortic rupture by 6–8 weeks of age [9].

At least two mechanisms have been proposed to explain the ability of MGP to inhibit vascular calcification: MGP binds to calcium ions, as well as to hydroxyapatite crystals, and may thereby directly inhibit crystal growth [17, 18, 19, 20, 21, 22]; MGP may also sequester bone morphogenetic protein (BMP)-2, BMP-4, and BMP-7 and reduce BMP signaling [23, 24, 25].

More than twenty ligands of the BMP family bind to heteromeric complexes of BMP type I and type II serine-threonine kinase receptors [26, 27]. BMP type II receptors phosphorylate BMP type I receptors, which in turn phosphorylate the cytosolic BMP effector proteins, Smads 1, 5, and 8 (Smad 1/5/8). Phosphorylated Smads 1/5/8 translocate to the nucleus together with Smad 4, where they activate specific targets, including the inhibitor of DNA binding (*Id*) genes [26]. BMPs are potent osteogenic factors that are required for osteoblast differentiation and bone formation [28].

BMP signaling has been implicated in intimal arterial wall calcification [7, 29]. Increased BMP-2 expression was observed in human atherosclerotic plaques [29]. Transgenic over-expression of BMP-2 in ApoE-deficient mice accelerated the development of intimal calcification [30]. Furthermore, BMP signaling promotes inflammation in atherosclerotic lesions, which indirectly promotes the development of intimal calcification [31]. Within calcified intimal vascular lesions, vascular smooth muscle cells (VSMCs) undergo accelerated proliferation, lose their smooth muscle contractile phenotype, and undergo transdifferentiation to an osteogenic phenotype [7, 32, 33]. *In vitro* studies demonstrated a relationship between BMP signaling and the expression of factors important for VSMC osteogenic transdifferentiation including runt-related transcription factor 2 (Runx2) [34, 35, 36].

The medial vascular calcification that develops in MGP-deficient mice is also characterized by a transdifferentiation of aortic VSMCs to osteogenic cells. This transdifferentiation is associated with both a loss of smooth muscle cell markers (including myocardin, α-smooth muscle actin (SMA), transgelin (tagln), and calponin), and an increase in osteogenic markers such as Runx2 and osteopontin (OPN) [37, 38, 39]. Runx2 is required for VSMC transdifferentiation and osteogenic activity [38, 40, 41]. The potential role of BMP signaling in the loss of VSMC phenotype, the increase in expression of osteogenic markers (Runx2 and OPN), and the medial vascular calcification associated with MGP deficiency is unknown.

The vascular endothelium provides a source of multipotent cells that contribute to vascular calcification in MGP-deficient mice, in a process termed endothelial-mesenchymal transition (EndMT) [42, 43]. Endothelial markers (VE-Cadherin and CD31) are increased and co-expressed with markers of multipotency (nanog, Oct 3/4, and sox2) prior to transitioning to mesenchymal cells that then express an osteogenic phenotype [42]. Depletion of MGP in cultured human aortic endothelial cells is associated with increased EndMT and calcification, effects that are enhanced by treatment with BMP-2, suggesting that BMP signaling is important for EndMT associated with vascular calcification [42].

To further investigate the role of BMP signaling in the loss of VSMC phenotype, EndMT, and medial vascular calcification associated with MGP deficiency, we studied the effects of inhibition of BMP signal transduction in MGP^-/-^ mice. We report that inhibition of BMP signaling using LDN-193189, a small molecule inhibitor of BMP type I receptor kinases [44], and ALK3-Fc, a recombinant protein that sequesters BMP ligands including BMP-2, BMP-4, and BMP-7, prevents vascular calcification associated with MGP deficiency. Long-term treatment of MGP^-/-^ mice with LDN-193189 improved survival. BMP signaling was essential for the development of osteogenic cells, EndMT, and VSMC calcification associated with MGP deficiency.

## Materials and Methods

### Chemicals and reagents

LDN-193189 (4-[6-(4-piperazin-1-ylphenyl)pyrazolo[1,5-a]pyrimidin-3-yl]quinoline) was synthesized as previously described [44], dissolved in water at a concentration of 0.5 mg/mL, and titrated to a pH of 5.5 with NaOH. Recombinant ALK3-Fc was provided by Acceleron Pharma Inc. (Cambridge, MA). OsteoSense-680 and ProSense-750 were obtained from PerkinElmer (Waltham, MA). Recombinant human BMP-2 was purchased from R&D Systems (Minneapolis, MN).

### Animals

This study was carried out in strict accordance with the recommendations in the Guide for the Care and Use of Laboratory Animals of the National Institutes of Health. Housing and all procedures involving mice described in this study, including survival studies, were specifically approved by the Institutional Animal Care and Use Committees of Massachusetts General Hospital (Subcommittee on Research Animal Care). All procedures were carried out with care to minimize suffering. For survival studies, the condition of the mice was checked twice daily. Any mice discovered to be with evidence of discomfort (manifested as immobility and/or an inability to feed) or respiratory distress (manifested by tachypnea) were promptly euthanized with CO_2_ gas. MGP^+/-^ mice were generated by Dr. Karsenty and colleagues [9]. Heterozygous mice were bred to obtain homozygous MGP^-/-^ mice, as well as MGP^+/+^ littermate control mice. Animals were maintained on a standard diet. MGP^-/-^ mice were treated intraperitoneally (i.p.) with LDN-193189 (2.5 mg/kg, once daily), ALK3-Fc (2 mg/kg every other day), or vehicle (water) starting on the first day of life and continuing for 28 days. Wild-type mice were treated with vehicle over the same time period. Aortas were harvested two hours after the last dose of LDN-193189, ALK3-Fc, or vehicle. Livers and lungs were harvested from vehicle-treated wild-type and MGP^-/-^ mice.

### Near-infrared imaging and quantification of aortic calcification and inflammation

Mice were injected via the tail vein with OsteoSense-680 and ProSense-750 (150 µl each) 24 hours before euthanasia, as described previously [45, 46]. Aortas were isolated and analyzed *ex vivo* by fluorescence reflectance imaging using an Odyssey Imaging System (LI-COR Biotechnology, Lincoln, NE) and software version 3.0.16 [31].

### Histology and Immunofluorescence

Aortas were embedded and cryopreserved in optimal cutting-temperature medium (Sakura Tissue-Tek, Zoeterwoude, Netherlands), and 6-µm sections were prepared [31]. To detect calcification, a solution of 2 g/dL Alizarin Red in distilled water (pH 4.2) was applied to aortic sections for 60 seconds, after which sections were washed with acetone and acetone-xylene (1:1) before mounting.

To detect macrophages in aortas, frozen tissue sections were fixed in cold 100% methanol and incubated with a monoclonal antibody specific for MAC-2 (Cedarlane, Burlington, NC) followed by Alexa Fluor 594-conjugated donkey anti-rat IgG antiserum (Jackson ImmunoResearch Laboratories, West Grove, PA). The location of nuclei was identified by staining with 4’,6-diamidino-2-phenylindole (DAPI).

### Preparation of mouse aortic vascular smooth muscle cells

Vascular smooth muscle cells (VSMCs) were isolated from aortas of MGP^-/-^ mice and wild-type littermate controls, as previously described [47]. Aortas were digested with Type 2 collagenase (175 U/mL, Worthington) and elastase (1.25 U/mL, Sigma) for 30 min, and the adventitial layer was removed. Aortas were further digested with collagenase and elastase for 60 min, and cells were plated and maintained in Dulbecco’s Minimum Essential Medium (DMEM, Invitrogen) supplemented with 10% fetal bovine serum (FBS, Invitrogen), 100 units/ml of penicillin, and 100 µg/ml of streptomycin at 37°C with 5% CO_2_. VSMC lineage was confirmed by immunocytochemistry using an antibody directed against α-smooth muscle actin (SMA, Sigma). Experiments with VSMCs were performed using cells that were passaged between 2–8 times. For gene expression experiments involving BMP-2 stimulation, cells were grown to 70% confluence followed by overnight serum starvation in DMEM with 0.1% fetal bovine serum.

### Measurement of gene expression by quantitative RT-PCR

Total RNA from aortas, livers, lungs, and cultured VSMCs was extracted by the phenol/guanidinium method [48]. Reverse transcription was performed using Moloney murine leukemia virus reverse transcriptase (Promega, Madison, WI, USA). A Mastercycler ep Realplex (Eppendorf, Hamburg, Germany) was used for real-time amplification and quantification of transcripts. Relative expression of target transcripts were normalized to levels of 18S ribosomal RNA, determined using the relative C_T_ method. Taqman gene expression assays were used to quantify mRNA levels encoding Id1 and Runx2. Quantitative PCR was performed with SYBR green for 18S, osteopontin (OPN), myocardin, SMA, transgelin (tagln), calponin, VE-cadherin, CD31, nanog, sox2, Oct 3/4, and MGP using the primer sequences in S1 Table.

### Immunoblot techniques

Aortas, livers, lungs, and VSMCs were homogenized in RIPA buffer containing protease and phosphatase inhibitors (Sigma). Tissue and cell lysates (20 µg/lane) were separated by SDS-PAGE, transferred to polyvinylidene difluoride (PVDF) membranes (GE Amersham Biosciences), and probed with antibodies specific for phosphorylated SMAD1/5 (P-SMAD1/5, rabbit monoclonal, Cell Signaling, catalog #9516S), total Smad 1 (mouse monoclonal, Life Span, catalog #LS-C75853), α-smooth muscle actin (mouse monoclonal, Sigma, catalog #A2547), or glyceraldehyde 3-phosphate dehydrogenase (GAPDH, rabbit monoclonal, Cell Signaling, catalog #5174S). Blots were incubated with horseradish peroxidase-conjugated anti-rabbit or anti-mouse IgG, and bound secondary antibodies were visualized by chemiluminescence (ECL Plus) and quantified using a VersaDoc Imaging System (BioRad, Hercules, CA).

### Depletion of MGP using siRNA

siRNA targeting MGP (siMGP) and scrambled control siRNA (siSC) were obtained from Dharmacon (SMARTpool, Thermo Scientific). Aortic VSMCs isolated from wild-type mice were transfected with siRNA using Lipofectamine RNAiMAX reagent, as described by the manufacturer (Life Technologies).

### Restoration of MGP using adenovirus-mediated gene transfer

Recombinant adenovirus directing expression of MGP (Ad.MGP) was constructed using a full length murine MGP cDNA (catalog # MMM1013–7514153, Open Biosytems) and the AdEasy system, as previously described [49]. A control adenovirus specifying GFP was purchased from Vector Biolabs (Ad.GFP, catalog # 1060). Ad.GFP and Ad.MGP were amplified in 293 cells. The virus was purified from cellular extracts using Adeno-X Mega Purification Kit (Clontech Laboratories, Mountain View, CA). Viral infectious forming units were quantified using the Adeno-X Rapid Titer Kit (Clontech Laboratories, Mountain View, CA). Aortic VSMCs isolated from MGP^-/-^ mice were infected with Ad.MGP or Ad.GFP at a multiplicity of infection of 10.

### Calcium Staining

To induce calcification in isolated VSMCs, cells were treated with DMEM supplemented with 10% FCS and 2 mM sodium phosphate, as previously described [38]. Cells were fixed in 10% formalin and incubated with von Kossa stain to detect calcification.

### Statistical analysis

Statistical analysis was performed using Graph Pad Prism 5.0 (GraphPad Software, La Jolla, CA). Data are reported as mean ± SEM, unless otherwise indicated. Two group comparisons of continuous variables were performed using the Student t test. For more than 2 group comparisons of continuous variables, analysis of variance (ANOVA) with post-hoc testing was employed. Survival was measured using the Kaplan-Meier method and compared using the log rank test. Cox proportional regression was used to determine the hazard ratio for mortality of LDN-193189-treated MGP^-/-^ mice compared to vehicle-treated mice. In all cases, a P<0.05 was considered to indicate statistical significance.

## Results

### BMP inhibition reduces vascular calcification and improves survival in MGP^-/-^ mice

**Figure 1:**
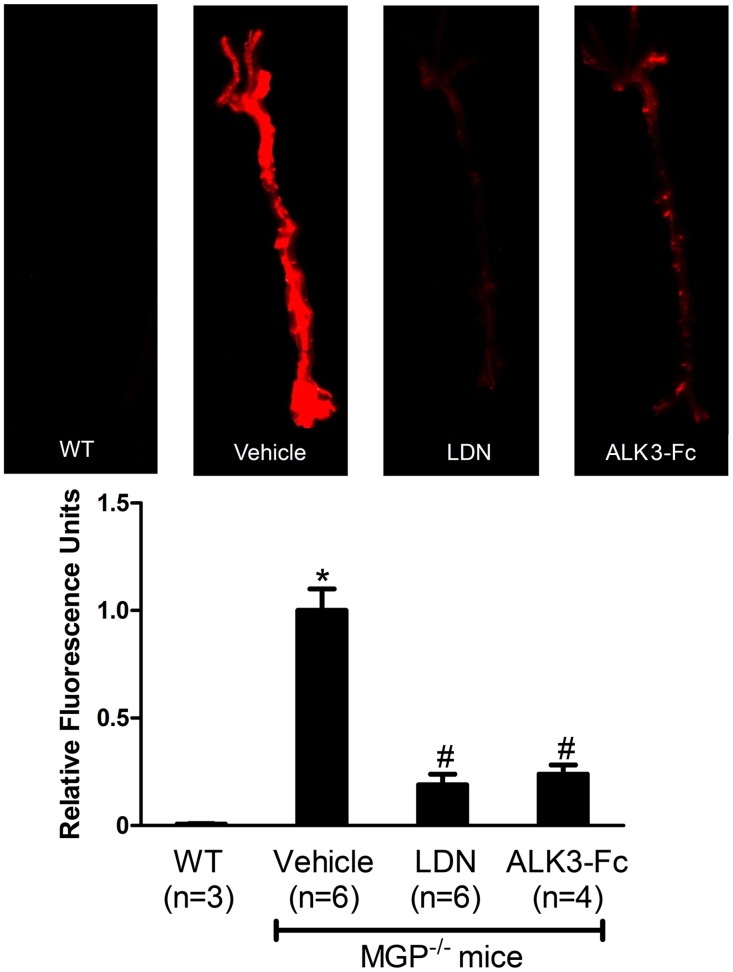
Pharmacologic inhibition of BMP signaling reduces osteogenic activity in the aortas of MGP-deficient mice. MGP^-/-^ mice were treated with vehicle, LDN-193189 (LDN), or ALK3-Fc starting at day 1 of life for 28 days. Wild-type mice (WT) were treated with vehicle. On day 27, OsteoSense-680 was injected via the tail vein. Aortas were harvested 24 hours later and imaged with near-infrared fluorescence (upper panel). Fluorescence intensities of the entire aorta normalized to vehicle-treated MGP^-/-^ mouse aortas were quantified (lower panel). Inhibition of BMP signaling reduced vascular calcification by 80% in MGP^-/-^ mice. * P<0.001 compared to vehicle-treated WT mice. # P<0.001 compared to vehicle-treated MGP^-/-^ mice.

**Figure 2:**
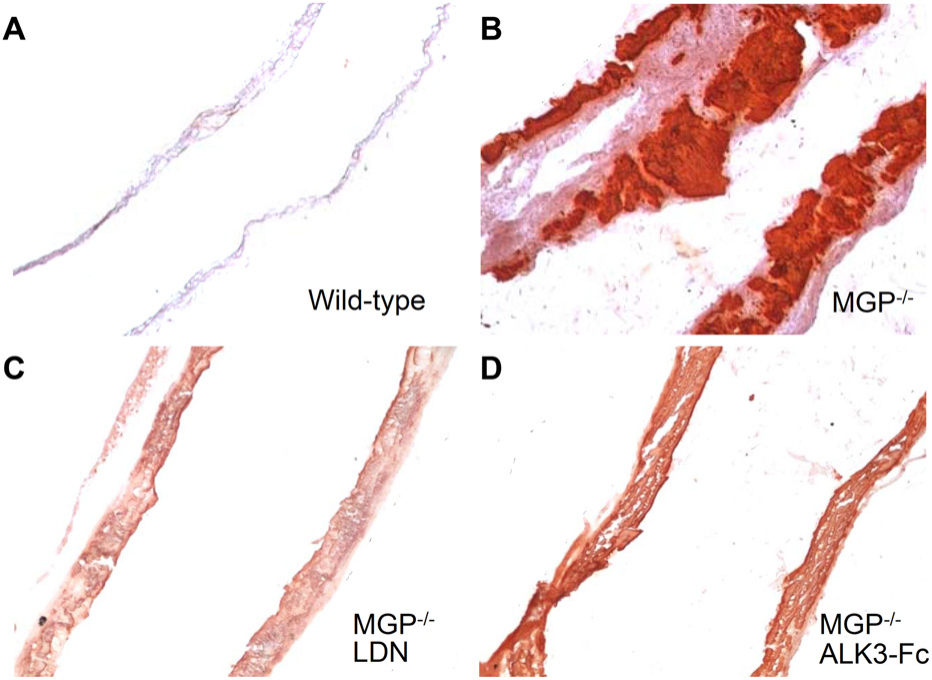
Vascular calcification associated with MGP deficiency is dependent on bone morphogenetic protein signaling. Aortas were harvested from mice at 28 days of age, sectioned, and stained for tissue calcium with Alizarin Red. Sections from wild-type (**A**) and MGP^-/-^ (**B-D**) mice were photographed at 100x magnification. Starting at day 1 of age, MGP^-/-^ mice were treated with i.p. injections of vehicle (**B**), LDN-193189 (LDN, 2.5 mg/kg daily, **C**), or ALK3-Fc (2 mg/kg every other day, **D**). Inhibition of BMP signaling reduced aortic calcification in MGP^-/-^ mice.

Calcification in the aortas of wild-type and MGP-deficient mice was quantified using OsteoSense-680, a near-infrared fluorescent bisphosphonate imaging probe that incorporates into the hydroxyapatite crystals of calcific lesions [45]. Aortas from wild-type mice exhibited little if any OsteoSense-680 labeling consistent with the absence of calcification (Fig. 1). A strong OsteoSense-680 signal was detected in the aortas and medium-sized arteries (e.g., carotid, subclavian, and iliac arteries) of 28-day-old MGP^-/-^ mice (Fig. 1). To confirm the presence of vascular calcification in the aortas of MGP^-/-^ mice, aortic tissue sections were stained with Alizarin Red. No aortic calcification was observed in MGP^-/-^ mice at 7 days of age or younger, but diffuse medial calcification was evident in aortas from 14-day-old MGP^-/-^ mice (data not shown), as previously reported [9]. In 28-day-old MGP^-/-^ mice, there was extensive aortic calcification with architectural distortion of the medial elastic layer. Calcification was not detected in the aortas of 28-day-old wild-type mice (Fig. 2A–B).

To determine whether the vascular calcification associated with MGP deficiency is dependent on BMP signaling, MGP^-/-^ mice were treated with intraperitoneal injections (i.p.) of LDN-193189 (2.5 mg/kg, once daily), ALK3-Fc (2 mg/kg every other day), or vehicle starting at day 1 of life and continuing for 28 days. Calcification was markedly reduced in the aortas of the two treatment groups compared to the vehicle-treated animals. Mice treated with inhibitors of BMP signaling exhibited an 80% reduction in aortic calcification compared to vehicle-treated MGP^-/-^ mice, as measured by incorporation of OsteoSense-680 (Fig. 1, P<0.001 for both LDN-193189 and ALK3-Fc versus vehicle). Furthermore, aortic tissue sections from MGP^-/-^ mice treated with either LDN-193189 or recombinant ALK3-Fc exhibited less Alizarin Red staining than did sections from vehicle-treated MGP^-/-^ mice (Fig. 2B–D).

**Figure 3:**
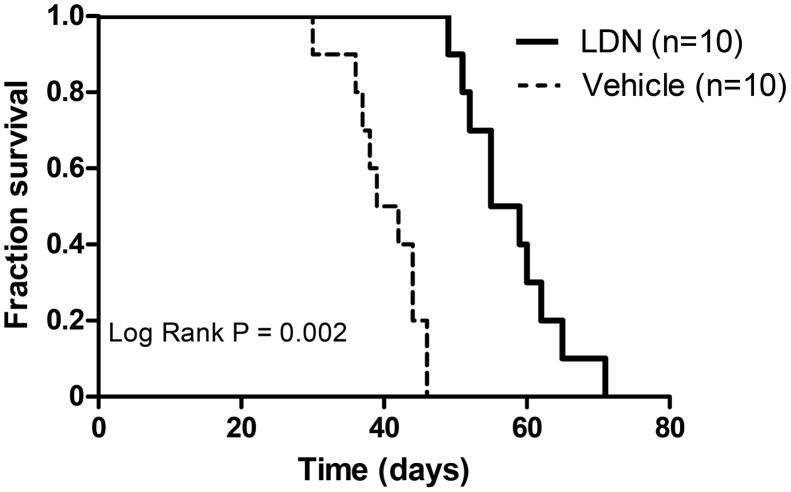
Inhibition of BMP signaling improves survival of MGP^-/-^ mice. MGP^-/-^ mice were treated once daily with LDN-193189 (LDN) or vehicle starting at day 1 of life. Kaplan-Meier survival was compared using the log rank test. MGP^-/-^ mice treated with LDN-193189 survived longer than vehicle-treated mice.

To determine whether inhibition of BMP signaling in MGP deficiency improves survival, MGP^-/-^ mice were treated with LDN-193189 (2.5 mg/kg i.p. daily) or vehicle starting at day 1 of age. Treatment with LDN-193189 improved survival (log rank P = 0.002, Fig. 3) with a Cox hazard ratio of 0.04 (95% CI 0.01–0.17). Vehicle-treated MGP^-/-^ mice had a median survival of 40 days, whereas LDN-193189-treated MGP^-/-^ mice exhibited a median survival of 57 days. LDN-193189-treated MGP^-/-^ mice also grew more rapidly than did vehicle-treated MGP^-/-^ mice (S1 Fig.). These results suggest that BMP signaling plays a critical role in the vascular calcification and mortality associated with MGP deficiency.

### MGP deficiency causes vascular calcification in the absence of inflammation

**Figure 4:**
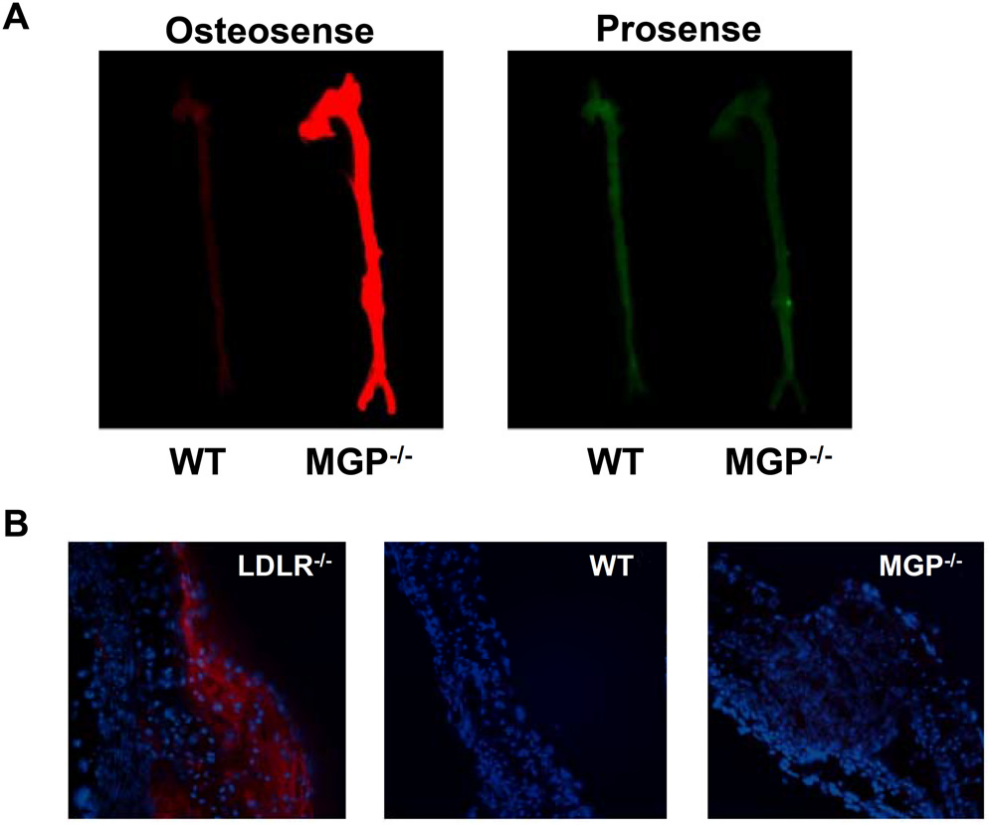
Vascular calcification associated with MGP deficiency occurs in the absence of vascular inflammation. (**A**) At 27 days of age, OsteoSense-680 and Prosense-750 were injected via the tail vein of wild-type (WT) and MGP^-/-^ mice. Aortas were harvested 24 hours later and imaged. Although aortas from MGP^-/-^ mice exhibited extensive vascular calcification, this calcification was not associated with increased macrophage activity. (**B**) Aortas were harvested from WT and MGP^-/-^ mice at 28 days of age, sectioned, and stained for macrophages with an antibody directed towards MAC-2. Aortas from LDLR^-/-^ mice on a high fat diet were used as a positive control. Nuclei were stained with DAPI. Similar to WT mice, macrophages were not detected by immunohistochemistry in the aortas of MGP^-/-^ mice.

In mouse models of atherosclerosis, we and others observed that intimal aortic calcification was associated with the presence of BMP-dependent vascular inflammation [31, 50]. To determine whether vascular calcification in MGP-deficient mice is associated with inflammation, MGP-deficient and wild-type mice were simultaneously injected with OsteoSense-680 and ProSense-750, the latter being a cathepsin-activated near-infrared fluorescent molecule that serves as a measure of macrophage activity in blood vessels [45]. Although the MGP^-/-^ aortas had a strong signal for calcification compared to wild-type aortas, there was no difference in macrophage activity between MGP^-/-^ and wild-type mice (Fig. 4A). To further assess for the potential presence of inflammation, aortic tissue sections were stained for MAC-2, a marker of macrophages (Fig. 4B). MAC-2 was not detected in aortic tissue sections from 28-day-old wild-type and MGP^-/-^ mice, compared to a high level of MAC-2 that was observed in aortic tissue sections obtained from LDLR^-/-^ mice fed a high fat diet, a murine model of atherosclerosis [31]. These findings indicate that MGP deficiency is a model of vascular calcification that occurs in the absence of vascular inflammation. The ability of LDN-193189 and ALK3-Fc to inhibit the vascular calcification observed in MGP^-/-^ mice is therefore independent of the potential effects of BMP inhibition on vascular inflammation.

### BMP signaling is not increased in MGP-deficient mice

**Figure 5:**
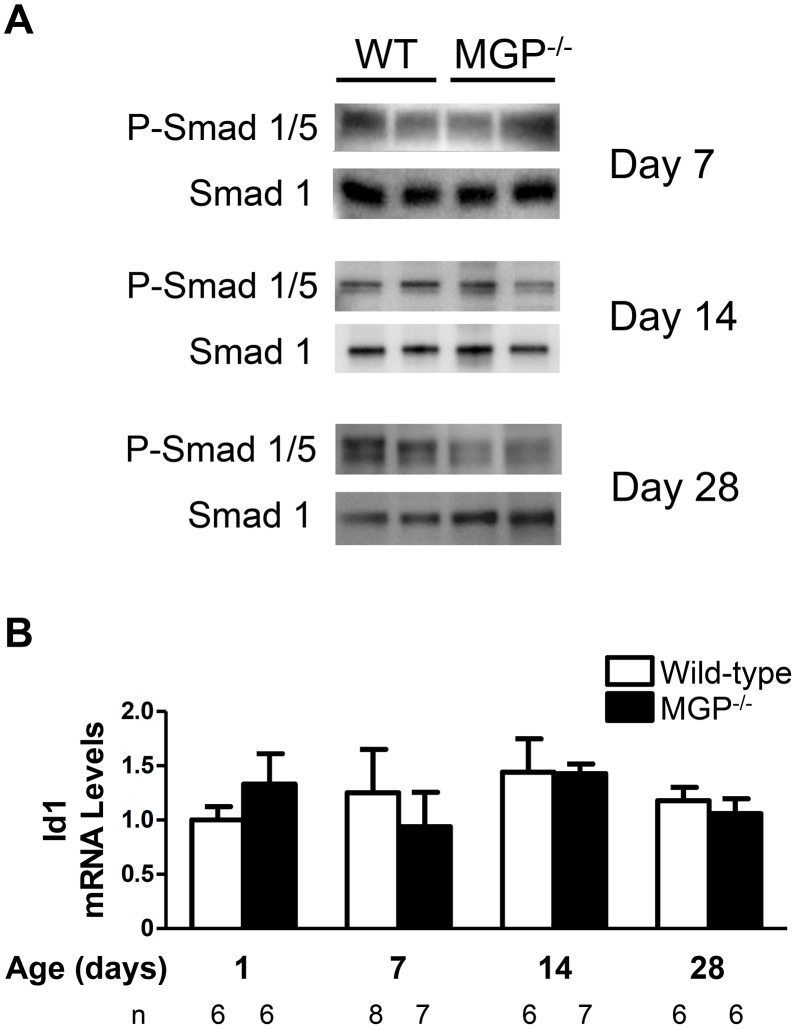
BMP signaling is not increased in aortas of MGP^-/-^ mice. (**A**) Protein lysates were isolated from the aortas of 7-, 14-, and 28-day-old WT and MGP^-/-^ mice. Each lane represents protein isolated from four pooled aortas. PVDF membranes were incubated with antibodies directed against phosphorylated Smad 1/5 (P-Smad 1/5) and total Smad 1. The ratio of P-Smad 1/5 to total Smad 1 was the same in aortas derived from WT and MGP^-/-^ mice. (**B**) RNA was isolated from aortas of WT and MGP^-/-^ mice at 1, 7, 14, and 28 days of (n = 6–8 in each group, as indicated). No difference in aortic Id1 mRNA levels was observed between MGP^-/-^ and WT mice.

It has been proposed that MGP inhibits vascular calcification by sequestering BMP ligands, thereby reducing BMP signaling [23, 24, 51]. To consider the possibility that MGP deficiency is associated with increased BMP signaling, levels of phosphorylated Smad 1/5 and total Smad 1 in the aortas of 7-, 14-, and 28-day-old wild-type and MGP^-/-^ mice were measured. Levels of phosphorylated Smad 1/5 normalized to total Smad 1 levels were not increased in the aortas of MGP^-/-^ mice compared to wild-type mice (Fig. 5A). To further assess the impact of MGP deficiency on aortic BMP signaling, Id1 mRNA levels in the aortas of wild-type and MGP^-/-^ mice at 1, 7, 14, and 28 days of age were measured (Fig. 5B). Id1 mRNA levels did not change over time in aortas from either wild-type or MGP^-/-^ mice, and Id1 mRNA levels did not differ between the two genotypes. To determine whether MGP deficiency alters BMP signaling in organs other than the aorta, Smad 1/5 phosphorylation and Id1 gene expression were measured in the livers and lungs of MGP^-/-^ and wild-type mice at 14 days of age. MGP gene expression was more than 100-fold greater in wild-type mouse lung than in liver (S2A Fig.). Across this broad range of MGP gene expression, Id1 mRNA levels and Smad 1/5 phosphorylation did not differ between MGP^-/-^ and wild-type mice (S2A-B Fig.). Taken together, these observations suggest that, although vascular calcification in MGP-deficient mice is dependent on BMP signaling, basal BMP signaling is not increased in the aortas, livers, and lungs of MGP^-/-^ mice compared to wild-type mice.

### MGP-deficiency does not alter BMP signaling in isolated vascular smooth muscle cells

**Figure 6:**
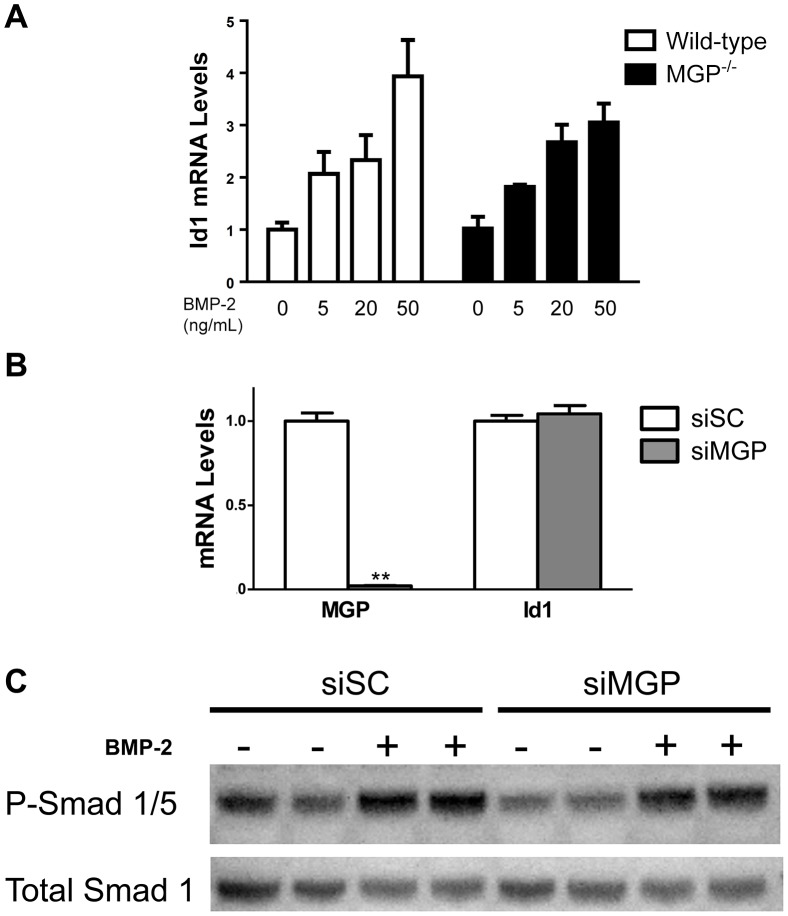
MGP deficiency does not alter basal BMP signaling or responsiveness to BMP-2 in VSMCs. (**A**) VSMCs were isolated from the aortas of wild-type and MGP^-/-^ mice. VSMCs were treated without or with recombinant human BMP-2 (for 2 hours at the indicated doses). Groups were compared using a 2-way ANOVA. Both WT and MGP^-/-^ VSMCs exhibited similar Id1 mRNA levels, both at baseline and in response to exogenous BMP-2. (**B**) Cultured aortic VSMCs from wild-type mice were transfected with either scrambled siRNA (siSC) or siRNA targeting MGP (siMGP) at 20 nM. RNA was isolated from cells after 4 days. siMGP decreased MGP mRNA levels in WT VSMCs by >95% compared with siSC-treated cells. However, depletion of MGP in WT VSMCs did not alter Id1 mRNA levels. **P<0.0001 compared to siSC-treated VSMCs. (**C**) VSMCs isolated from wild-type mice were treated with 20 nM of either scrambled siRNA (siSC) or siRNA specific for MGP (siMGP). Cells were incubated with or without BMP-2 (20 ng/mL) for 1 h prior to protein harvest. Western blots were probed with antibodies specific for phosphorylated Smad 1/5 (P-Smad 1/5) and total Smad 1. Depletion of MGP in WT VSMCs did not alter the ratio of P-Smad 1/5 levels to total Smad 1 levels, both at baseline and in response to exogenous BMP-2.

Although increased BMP signaling was not observed in the whole aortas of MGP^-/-^ mice, it was possible that individual cells types within the vasculature such as VSMCs might exhibit increased BMP signaling in the absence of MGP [23, 24, 51]. To determine whether MGP deficiency enhances the response of VSMCs to BMP ligands, VSMCs were isolated from the aortas of wild-type and MGP^-/-^ mice and were incubated in the presence or absence of BMP-2. At baseline, Id1 mRNA levels did not differ between wild-type and MGP^-/-^ VSMCs (Fig. 6A). Incubation with varying concentrations of BMP-2 increased Id1 mRNA to similar levels in wild-type and MGP^-/-^ VSMCs. In complementary experiments, siRNA specific for MGP affected neither basal Id1 gene expression nor basal or BMP-2-stimulated Smad 1/5 phosphorylation in isolated aortic VSMCs derived from wild-type mice (Fig. 6B–C). These results demonstrate that MGP deficiency does not augment the sensitivity of isolated VSMCs to BMP signaling.

### BMP signaling does not cause the loss of VSMC phenotype associated with MGP deficiency

**Figure 7:**
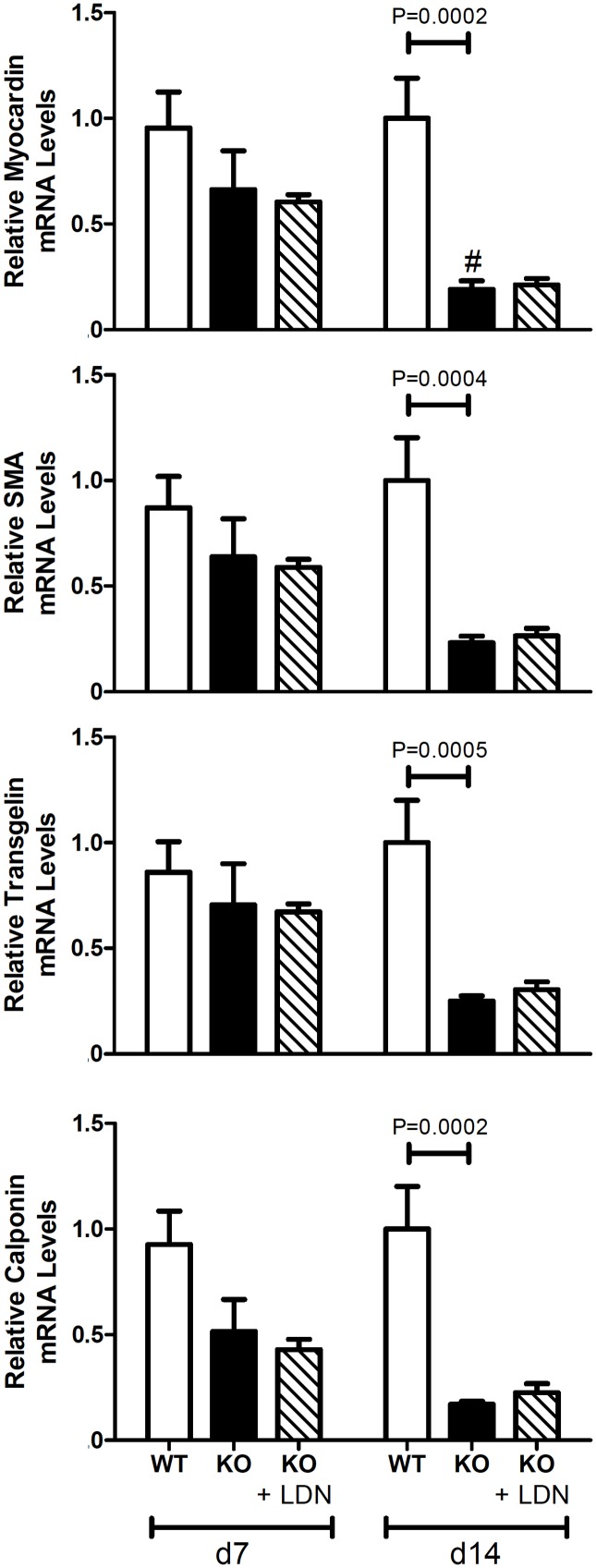
Aortic expression of VSMC markers in wild-type and MGP^-/-^ mice. RNA was isolated from aortas of WT and MGP^-/-^ mice and from LDN-193189-treated MGP^-/-^ mice at 7 and 14 days of age (n = 4–8 in each group). Levels of mRNAs encoding myocardin, α smooth muscle actin (SMA), transgelin, and calponin are depicted. The aortas of 14-day-old MGP^-/-^ mice have decreased expression of VSMC markers compared to WT mice. Treatment with LDN-193189 did not restore the expression of VSMC markers to WT levels. # P<0.05 compared to 7-day-old MGP^-/-^ mice.

Vascular calcification in MGP-deficient mice is associated with a loss of VSMC phenotype [37, 41]. To determine whether this loss of phenotype associated with MGP deficiency is dependent on BMP signaling, we measured levels of mRNAs encoding VSMC markers in aortas harvested from 7- and 14-day-old wild-type and MGP^-/-^ mice, in the absence and presence of LDN-193189. In wild-type mice, levels of four VSMC markers (myocardin, SMA, tagln, and calponin) did not differ in aortas of 7- and 14-day-old animals (Fig. 7). In contrast, expression of myocardin was less in the aortas of 14-day-old MGP^-/-^ mice than in those of 7-day-old MGP^-/-^ mice, and the expression of SMA, tagln, and calponin tended to decrease with age (P = 0.06, 0.07, and 0.06, respectively). Levels of all four VSMC markers were less in aortas of MGP^-/-^ mice than in those of wild-type mice at 14 days of age. Consistent with decreased SMA mRNA levels, we observed that SMA protein levels were less in aortas of MGP^-/-^ mice than in those of wild-type mice (S3 Fig.). These results show that VSMC markers decrease with age in MGP^-/-^ mice but not in wild-type mice, consistent with a loss of VSMC phenotype.

To determine whether BMP signaling is necessary for the loss of VSMC phenotype in the aortas of MGP^-/-^ mice, we measured the levels of myocardin, SMA, tagln, and calponin mRNA in the aortas of MGP^-/-^ mice treated with LDN-193189 beginning at 1 day of age. Inhibition of BMP signaling with LDN-193189 did not normalize expression of the markers of VSMC phenotype in aortas of 14-day-old MGP^-/-^ mice compared to wild-type mice (Fig. 7). These results suggest that BMP signaling does not cause the loss of VSMC phenotype observed in the aortas of MGP^-/-^ mice.

### BMP signaling is required for the induction of osteogenic markers in the aortas of MGP^-/-^ mice

**Figure 8:**
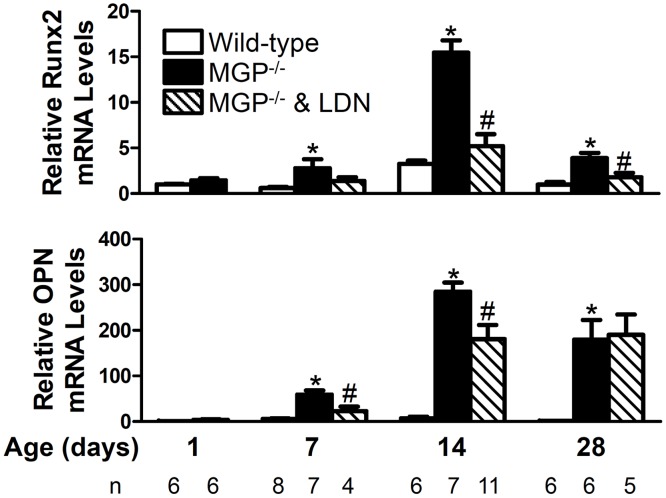
BMP signaling is required for the increased aortic expression of osteogenic markers associated with MGP deficiency. RNA was isolated from aortas of WT and MGP^-/-^ mice at 1, 7, 14, and 28 days of age and from LDN-193189-treated MGP^-/-^ mice at 7, 14, and 28 days of age (n = 4–11 in each group, as indicated). Expression of genes encoding Runx2 and osteopontin (OPN) was measured. MGP^-/-^ mice had increased levels of aortic Runx2 and OPN mRNA compared to WT mice. Treatment of MGP^-/-^ mice with LDN-193189 reduced aortic Runx2 and OPN mRNA levels. * P<0.001 compared to WT mice of same age. # P<0.05 compared to age-matched MGP^-/-^ mice treated with vehicle.

The medial vascular calcification that develops in MGP-deficient mice is characterized by a phenotypic switch of aortic VSMCs to osteogenic cells. To investigate the effects of BMP inhibition on aortic osteogenic transdifferentiation, we measured aortic levels of mRNAs encoding osteogenic markers, Runx2 and OPN, in 1-, 7-, 14-, and 28-day-old MGP^-/-^ and wild-type mice, both in the absence and presence of LDN-193189 (Fig. 8). Aortic Runx2 mRNA levels did not change over time in wild-type mice. In contrast, aortic expression of Runx2 increased with age in the MGP^-/-^ mice, peaking at 14 days. Runx2 gene expression was greater in MGP^-/-^ aortas at 7, 14, and 28 days of age than in aortas from age-matched wild-type mice. Similar to Runx2, aortic OPN gene expression did not change over time in wild-type mice but increased with time in MGP^-/-^ mice, peaking at 14 days. OPN mRNA levels were greater in MGP^-/-^ aortas at 7, 14, and 28 days of age than in aortas from wild-type mice.

To determine whether increased aortic Runx2 and OPN in MGP-deficient mice is dependent on BMP signaling, we examined the expression of mRNAs encoding Runx2 and OPN in aortas of MGP^-/-^ mice treated with LDN-193189. To confirm that LDN-193189 was able to inhibit aortic BMP signaling, levels of Id1 mRNA were measured. Treatment with LDN-193189 reduced Id1 gene expression by 70–80% in the aortas of 7-, 14-, and 28-day old MGP^-/-^ mice (S4 Fig.). Inhibition of BMP signaling was associated with a 70% and 50% reduction in aortic Runx2 mRNA levels at 14 and 28 days of age, respectively (Fig. 8). Treatment with LDN-193189 reduced aortic OPN mRNA levels in 7- and 14-day-old MGP^-/-^ mice by 60% and 40%, respectively. These results suggest that the observed increase in Runx2 and OPN in the aortas of MGP-deficient mice is dependent, at least in part, on BMP signaling.

### BMP signal transduction is required for VSMC calcification observed in MGP deficiency

**Figure 9:**
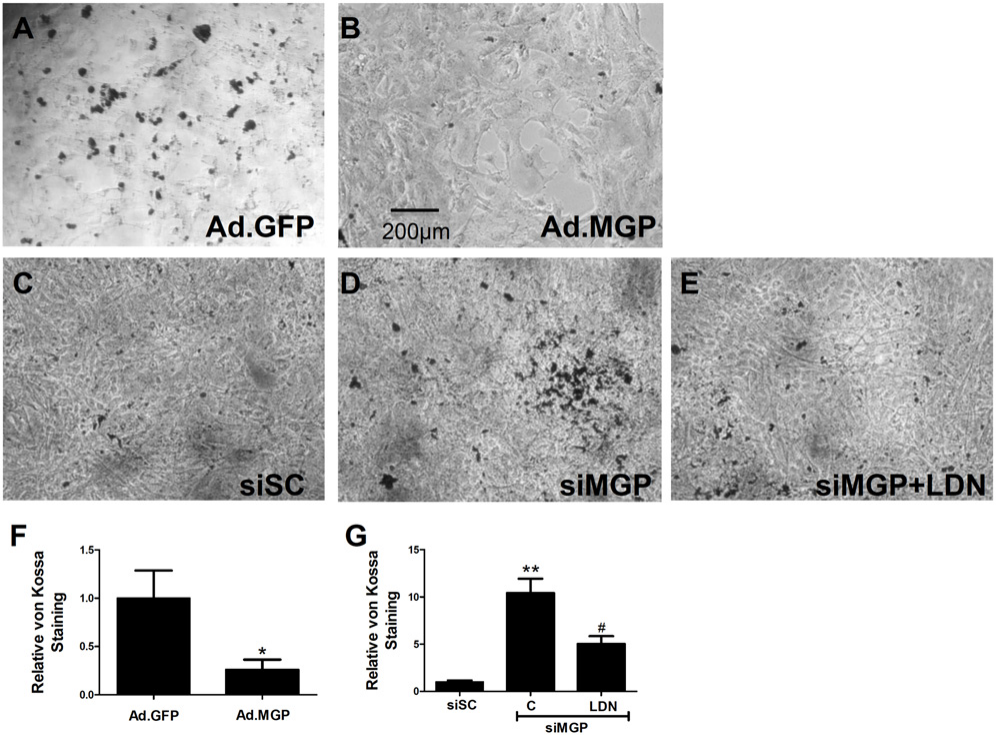
Restoration of MGP levels decreases calcification of MGP^-/-^ vascular smooth muscle cells while siRNA-mediated depletion of MGP increases calcification of wild-type vascular smooth muscle cells in a BMP-dependent manner. Cultured aortic VSMCs isolated from MGP^-/-^ mice were infected with either (**A**) a control adenovirus (Ad.GFP) or (**B**) an adenovirus expressing MGP (Ad.MGP) at a multiplicity of infection of 10 and placed in DMEM supplemented with 10% FBS and 2 mM sodium phosphate. Cultured aortic VSMCs isolated from wild-type mice were transfected with either (**C**) scrambled siRNA (siSC) or (**D & E**) siRNA targeting MGP (siMGP) at 20 nM and placed in DMEM supplemented with 10% FBS and 2 mM sodium phosphate. Cells were also treated without (**C & D**) or with (**E**) 100 nM LDN-193189 (LDN). Cells were stained after 7 days using the von Kossa method. Serial fields of view were photographed for each condition and von Kossa stain was quantified using image J software after background subtraction (**F & G**). In (**F**), *P = 0.03 compared to Ad.GFP. In (**G**), **P<0.0001 compared to siSC-treated cells. #P = 0.0003 compared to siMGP + control. Restoration of MGP expression reduced phosphate-induced calcification of MGP^-/-^ VSMCs, while depletion of MGP increased calcification of WT VSMCs and this calcification was partially inhibited by treatment with LDN-193189.

The process of vascular calcification involves the initial loss of VSMC phenotype followed by the development of osteogenic cells and subsequent calcification [38]. Although we observed that BMP inhibition did not prevent loss of VSMC phenotype in the aortas of MGP^-/-^ mice, BMP signaling was required for the induction of osteogenic cells. We next investigated whether calcification of MGP-deficient VSMCs is dependent on BMP signaling *in vitro*. Calcification was induced in isolated VSMCs by growing the cells in DMEM supplemented with 10% FCS and 2 mM sodium phosphate [38]. Expression of MGP in MGP^-/-^ VSMCs using an adenovirus vector reduced calcification by 70%, as detected by von Kossa stain, compared with adenovirus control-treated cells (Fig. 9A–B, F). Treatment of wild-type VSMCs with siRNA directed against MGP (siMGP) increased calcification compared to treatment with control scrambled siRNA (siSC) (Fig. 9C–D). These results indicate that, similar to *in vivo* findings, MGP expression modulates the calcification occurring in cultured VSMCs treated with high phosphate-containing media. Treatment with LDN-193189 inhibited the calcification induced by siMGP in wild-type VSMCs by approximately 50% (P = 0.0003; Fig. 9D–E, 9G). Taken together, these observations suggest that MGP inhibits calcification of isolated VSMCs and that calcification of MGP-deficient VSMCs is, at least in part, dependent on BMP signal transduction.

### BMP signal transduction is required for the increased endothelial-mesenchymal transition observed in MGP-deficient mice

**Figure 10:**
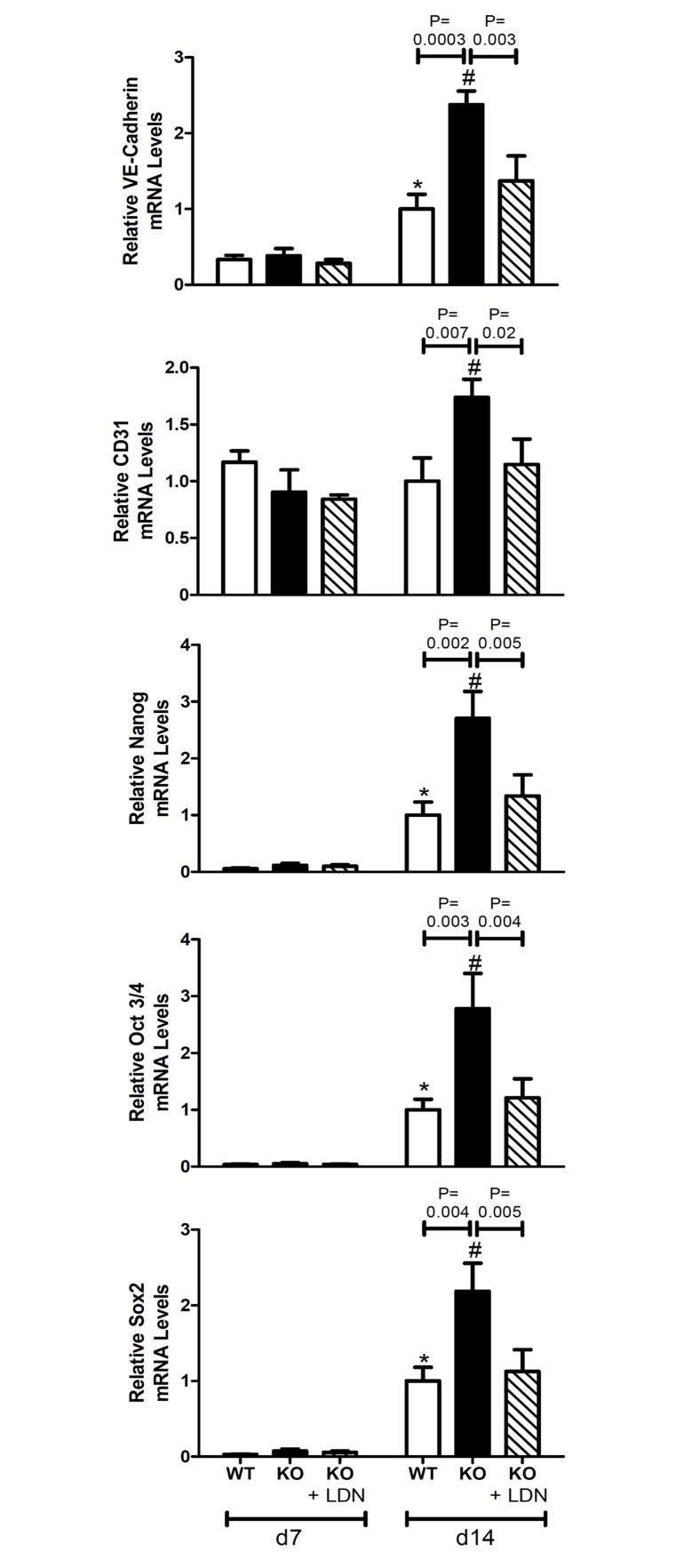
Aortic expression of endothelial and multipotency markers in wild-type and MGP^-/-^ mice. RNA was isolated from aortas of WT and MGP^-/-^ mice and from LDN-193189-treated MGP^-/-^ mice at 7 and 14 days of age (n = 4–8 in each group). Levels of mRNAs encoding endothelial markers (VE-Cadherin and CD31) and multipotency markers (nanog, Oct 3/4, and Sox2) are depicted. The aortas of 14-day-old MGP^-/-^ mice have increased endothelial and multipotency markers compared to WT mice. Treatment with LDN-193189 normalized the endothelial and multipotency markers to WT levels in MGP^-/-^ mice. * P ≤ 0.01 compared to 7-day-old WT mice. # P<0.05 compared to 7-day-old MGP^-/-^ mice.

A recent study identified endothelial cells in the aortas of MGP-deficient mice as a source of multipotent progenitor cells which subsequently give rise to osteogenic mesenchymal cells [42]. To investigate the potential role of BMP signaling in the induction of EndMT, levels of mRNAs encoding endothelial markers (VE-cadherin and CD31) and multipotency markers (nanog, Oct 3/4, and sox2) were measured in aortas harvested from 7- and 14-day-old wild-type and MGP^-/-^ mice treated with LDN-193189 or vehicle. In the absence of BMP inhibition, levels of mRNAs ecoding VE-cadherin and multipotency markers were greater in aortas of 14-day-old compared with 7-day-old wild-type mice (Fig. 10). Endothelial and multipotency markers were greater in the aortas of 14-day-old MGP^-/-^ mice than in those of 7-day-old MGP^-/-^ mice. The expression of endothelial and multipotency markers was greater in the aortas of 14-day-old MGP^-/-^ mice compared with age-matched wild-type mice, suggesting that the enhanced EndMT observed in MGP^-/-^ mice begins after the first week of life. Treatment with LDN-193189 reduced the expression of endothelium and multipotency markers in the aortas of 14-day-old MGP^-/-^ mice to the levels seen in wild-type mice. These observations indicate that BMP signaling is required for the increased EndMT associated with MGP deficiency.

## Discussion

In this study, we identified an essential role for basal BMP signaling in the development of medial vascular calcification associated with MGP deficiency. Inhibition of basal BMP signaling prevented vascular calcification and improved survival in MGP^-/-^ mice. Although the loss of aortic VSMC phenotype observed in MGP deficiency was not dependent on BMP signaling, the subsequent expression of osteogenic factors in the aortas of MGP^-/-^ mice was dependent on BMP signaling and our *in vitro* data implicate BMP signaling as a cause of calcification in isolated VSMCs. BMP signaling was also required for the increased EndMT associated with MGP deficiency. Thus, this study has identified BMP-dependent and BMP-independent aspects of vascular cell phenotype and calcification associated with MGP deficiency.

Prior studies demonstrated a role for BMP signaling in the development of atherosclerosis and intimal calcification [31, 50]. Transgenic over-expression of BMP-2 under the direction of the SMA promoter accelerated intimal calcification in ApoE-deficient mice with atherosclerosis [30]. Furthermore, inhibition of BMP signaling in low-density lipoprotein receptor (LDLR)-deficient mice fed a high-fat diet reduced intimal calcification [31]. This previous work demonstrated a role for BMP signaling in regulating LDL levels and macrophage recruitment to the vessel wall [31]. However, the MGP^-/-^ mouse model differs from atherosclerotic mouse models in that calcification develops in the medial layer of arteries and occurs in the absence of vascular inflammation. Two inhibitors of BMP signaling with different modes of action, LDN-193189 (which targets the BMP type I receptor) and ALK3-Fc (which targets BMP ligands), both inhibited vascular calcification. Inhibition of BMP signaling also improved survival and growth in MGP-deficient mice. These results identify an important role for BMP signaling in medial vascular calcification and demonstrate that inhibition of BMP signaling can ameliorate calcification of the medial arterial layer that occurs in the absence of inflammation.

A previous report suggested that levels of phosphorylated BMP-responsive Smads are greater in the aortas of MGP^-/-^ mice than in those of wild-type mice [50]. In this study, we did not observe increased aortic levels of phosphorylated Smad 1/5 or Id1 mRNA in MGP^-/-^ compared to wild-type mice. We also did not detect a difference in basal BMP signaling in the livers (expressing low levels of MGP in wild-type mice) and lungs (expressing higher levels of MGP in wild-type mice) of MGP^-/-^ and wild-type mice. In studies of aortic VSMCs isolated from wild-type and MGP^-/-^ mice, there was no difference in Smad 1/5 phosphorylation or Id1 gene expression at baseline or after stimulation with BMP-2. Furthermore, depletion of MGP from wild-type aortic VSMCs using siRNA did not alter levels of Smad 1/5 phosphorylation or Id1 mRNA. Similar to our *in vivo* findings, inhibition of basal BMP signaling in aortic VSMCs depleted of MGP with siRNA prevented the calcification induced by high phosphate conditions. Taken together, the findings suggest that MGP deficiency results in vascular calcification via a mechanism that requires basal, but not increased BMP signaling.

MGP appears to have an important role in the regulation of VSMC phenotype *in vivo* [21, 38, 41, 42]. Speer and colleagues reported that VSMCs in MGP-deficient mice lose their smooth muscle phenotype and transdifferentiate into osteogenic cells [41]. This study confirmed that MGP deficiency was associated with reduced aortic levels of VSMC markers. However, inhibition of BMP signaling starting at day 1 of life did not prevent the loss of VSMC phenotype in MGP^-/-^ mice. These results suggest that, in the absence of MGP, the loss of VSMC phenotype occurs via a mechanism that is independent of BMP signaling.

To investigate the role of BMP signaling in the induction of the osteogenic phenotype in the vasculature of MGP^-/-^ mice, we examined the impact of LDN-193189 on aortic expression of Runx2 and OPN. Runx2 is an essential regulator of the osteogenic differentiation of vascular cells [52], and VSMC-specific deficiency of Runx2 protects ApoE-deficient mice from high-fat diet-induced vascular calcification [53]. OPN is a marker of osteogenesis and vascular calcification in MGP-deficient mice [54]. Compared to wild-type mice, MGP^-/-^ mice had increased aortic Runx2 and OPN mRNA levels beginning at 7 days of age. Treatment of MGP^-/-^ mice with LDN-193189 decreased aortic Runx2 mRNA levels in 14- and 28-day-old mice, and treatment with the BMP inhibitor reduced aortic OPN mRNA levels in 7- and 14-day-old mice. These results suggest that inhibition of BMP signaling reduces vascular calcification in MGP^-/-^ mice by preventing development of an osteogenic phenotype.

In MGP^-/-^ mice, endothelial cells undergo EndMT prior to differentiation into osteogenic cells [42]. MGP deficiency is associated with increased aortic expression of markers of endothelial cells (VE-cadherin and CD31) and multipotent cells (Nanog, Oct 3/4, Sox2). Treatment of MGP^-/-^ mice with LDN-193189 reduced aortic expression of endothelial and multipotency markers to levels seen in wild-type mice. These observations suggest that the EndMT associated with MGP deficiency is dependent on BMP signaling. The results are complementary to the findings of Yao et al. who demonstrated that BMP signaling augments EndMT in cultured MGP-deficient human aortic endothelial cells [42].

BMP inhibition delayed, but did not prevent, premature death of MGP-deficient mice. On necropsy, some aortic calcification was evident in the aortas of treated mice. It is possible that once daily dosing of LDN-193189 may provide only intermittent inhibition of vascular BMP signaling, delaying but not fully preventing vascular calcification. Alternatively, because treatment with LDN-193189 did not prevent loss of the VSMC phenotype, it is possible that less efficient, BMP-independent pathways may contribute to subsequent osteogenesis.

In summary, our work has identified the BMP signaling pathway as an important mediator of calcification of the vascular media and inhibition of BMP signaling can reduce vascular calcification and improve survival in MGP^-/-^ mice. MGP deficiency does not increase aortic BMP signaling; basal BMP signaling is sufficient to permit vascular calcification. While BMP signaling is not required for the loss of VSMC phenotype associated with MGP deficiency, the development of an osteogenic phenotype and subsequent calcification of VSMCs is BMP signaling-dependent. The increased endothelial-to-mesenchymal transition observed in MGP deficiency also depends on BMP signaling. Because BMP signaling appears to have a critical role in several steps in the pathogenesis of medial artery calcification, inhibition of the BMP pathway may prove to be an effective approach for the prevention or treatment of this vascular disease.

## Supporting Information

S1 Table Primer sequences. The forward and reverse primer sequences used for gene expression analysis in this study.
(DOC)

S1 Fig Treatment with LDN-193189 improves growth of MGP^-/-^ mice. MGP^-/-^ mice were treated with daily i.p. injections of LDN-193189 (LDN) or vehicle starting at day 1 of life and weighed weekly. Increased body weight was detected in LDN-193189-treated compared with vehicle-treated MGP^-/-^ mice beginning at 21 days. Data are presented as mean ± standard deviation (n = 10 in each group). *P<0.001 compared to vehicle-treated group of same age.
(TIF)

S2 Fig BMP signaling does not differ in the livers and lungs of MGP^-/-^ mice compared to wild-type mice. (**A**) MGP mRNA levels were more than 100-fold greater in the lungs compared with the livers of wild-type mice (left panel). *P<0.001 compared to liver MGP mRNA levels. No difference in Id1 mRNA levels was detected between WT and MGP^-/-^ mice, both in the livers and lungs (n = 6 in each group, right panel). (**B**) Smad 1/5 phosphorylation (P-Smad 1/5) and total Smad 1 levels were measured in the livers and lungs of wild-type (n = 6) and MGP^-/-^ mice (n = 6) at 14 days of age. There was no difference in the ratio of P-Smad 1/5 to total Smad 1 protein levels in WT and MGP^-/-^ mice, in either liver or lung.
(TIF)

S3 Fig MGP deficiency is associated with decreased levels of smooth muscle actin. Protein lysates were harvested from aortas of WT and MGP^-/-^ mice. PVDF membranes were treated with antibodies directed against SMA and GAPDH. Aortas from MGP^-/-^ mice have reduced SMA protein levels compared to those of WT mice.
(TIF)

S4 Fig LDN-193189 inhibits aortic BMP signaling in MGP^-/-^ mice. RNA was isolated from aortas of MGP^-/-^ mice treated with either LDN-193189 or vehicle at 7, 14, and 28 days of age (n = 4–11 in each group, as indicated). Treatment of MGP^-/-^ mice with LDN-193189 reduced aortic Id1 mRNA levels by 70–80%. # P<0.05 compared to age-matched MGP^-/-^ mice treated with vehicle.
(TIF)
