# Supplementary material for: Inhibition of Bone Morphogenetic Protein Signal Transduction Prevents the Medial Vascular Calcification Associated with Matrix Gla Protein Deficiency
Source: PLoS One. 2015 Jan 20;10(1):e0117098. doi: 10.1371/journal.pone.0117098 (PMC4300181; doi:10.1371/journal.pone.0117098)
Supplement: S1 Table — The forward and reverse primer sequences used for gene expression analysis in this study. (DOC) [file pone.0117098.s001.doc]

**Supplementary Table 1: Primer sequences** utilized for gene expression analysis.

| **Gene Name** | **Forward Primer** | **Reverse Primer** |
| --- | --- | --- |
| 18S | 5’-CGGCTACCACATCCAAGGAA-3’ | 5’-GCTGGAATTACCGCGGCT-3’ |
| Osteopontin | 5’-AGCAAGAAACTCTTCCAAGCAA-3’ | 5’-GTGAGATTCGTCAGATTCATCCG-3’ |
| Myocardin | 5’- GATGGGCTCTCTCCAGATCAG-3’ | 5’-GGCTGCATCATTCTTGTCACTT-3’ |
| SMA | 5'-CCCAGACATCAGGGAGTAATGG-3' | 5'-TCTATCGGATACTTCAGCGTCA-3' |
| Transgelin | 5'-GTGTGATTCTGAGCAAATTGGTG-3' | 5'-ACTGCTGCCATATCCTTACCTT-3' |
| Calponin | 5'-TCTGCACATTTTAACCGAGGTC-3' | 5'-GCCAGCTTGTTCTTTACTTCAGC-3' |
| VE-Cadherin | 5'-CCACTGCTTTGGGAGCCTT-3' | 5'-GGCAGGTAGCATGTTGGGG-3' |
| CD31 | 5'-ACGCTGGTGCTCTATGCAAG-3' | 5'-TCAGTTGCTGCCCATTCATCA-3' |
| Nanog | 5'-CACAGTTTGCCTAGTTCTGAGG-3' | 5'-GCAAGAATAGTTCTCGGGATGAA-3' |
| Sox2 | 5'-GCGGAGTGGAAACTTTTGTCC-3' | 5'-GGGAAGCGTGTACTTATCCTTCT-3' |
| Oct 3/4 | 5'-AGAGGATCACCTTGGGGTACA-3' | 5'-CGAAGCGACAGATGGTGGTC-3' |
| MGP | 5'-AGCCCAAAAGAGAGTCCAGGA-3' | 5'-CTGCCTGAAGTAGCGGTTGTA-3' |
