# Supplementary figures and images for: Inhibition of Bone Morphogenetic Protein Signal Transduction Prevents the Medial Vascular Calcification Associated with Matrix Gla Protein Deficiency
Source: PLoS One. 2015 Jan 20;10(1):e0117098. doi: 10.1371/journal.pone.0117098 (PMC4300181; doi:10.1371/journal.pone.0117098)

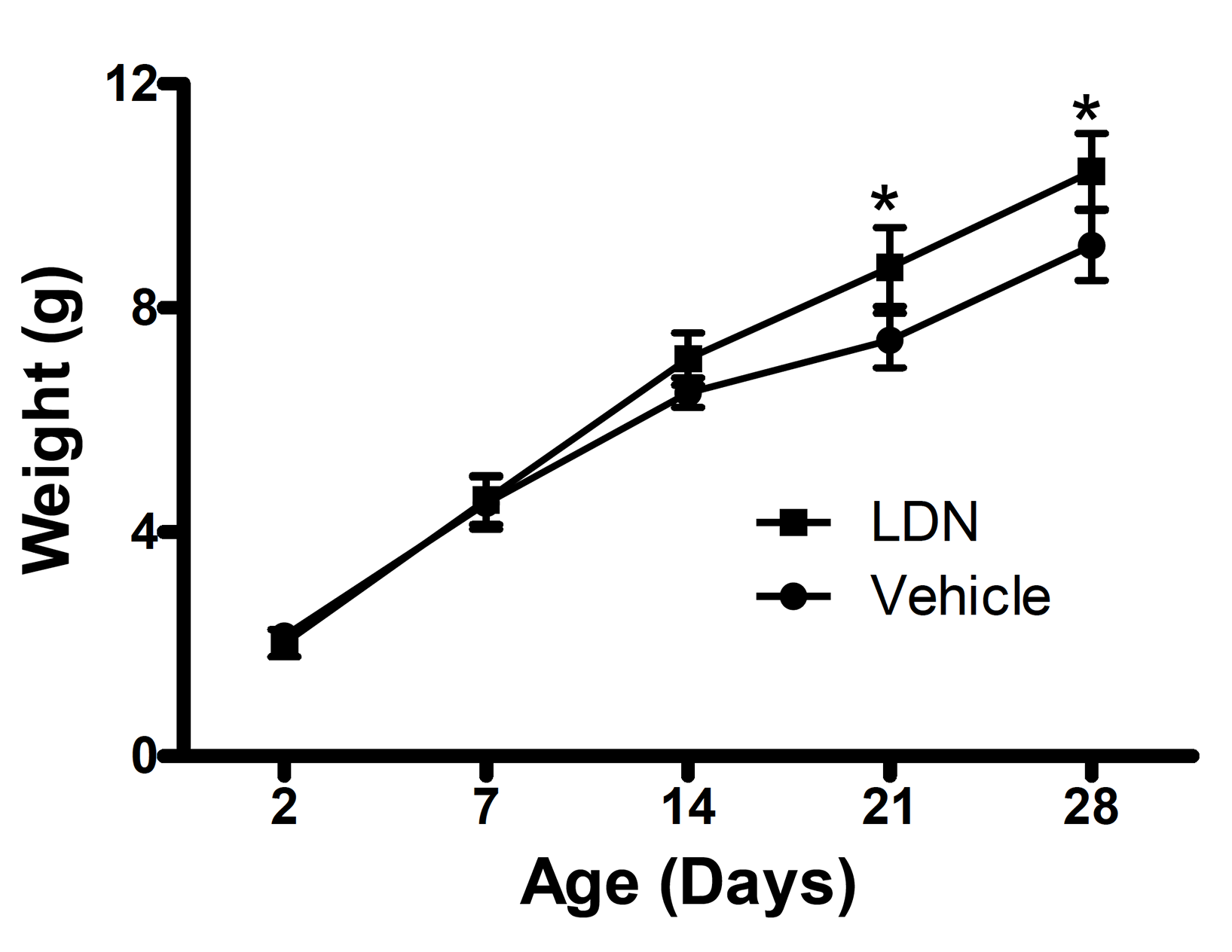

Supplement: S1 Fig — MGP-/- mice were treated with daily i.p. injections of LDN-193189 (LDN) or vehicle starting at day 1 of life and weighed weekly. Increased body weight was detected in LDN-193189-treated compared with vehicle-treated MGP-/- mice beginning at 21 days. Data are presented as mean ± standard deviation (n = 10 in each group). *P<0.001 compared to vehicle-treated group of same age. (TIF) [file pone.0117098.s002.tif]

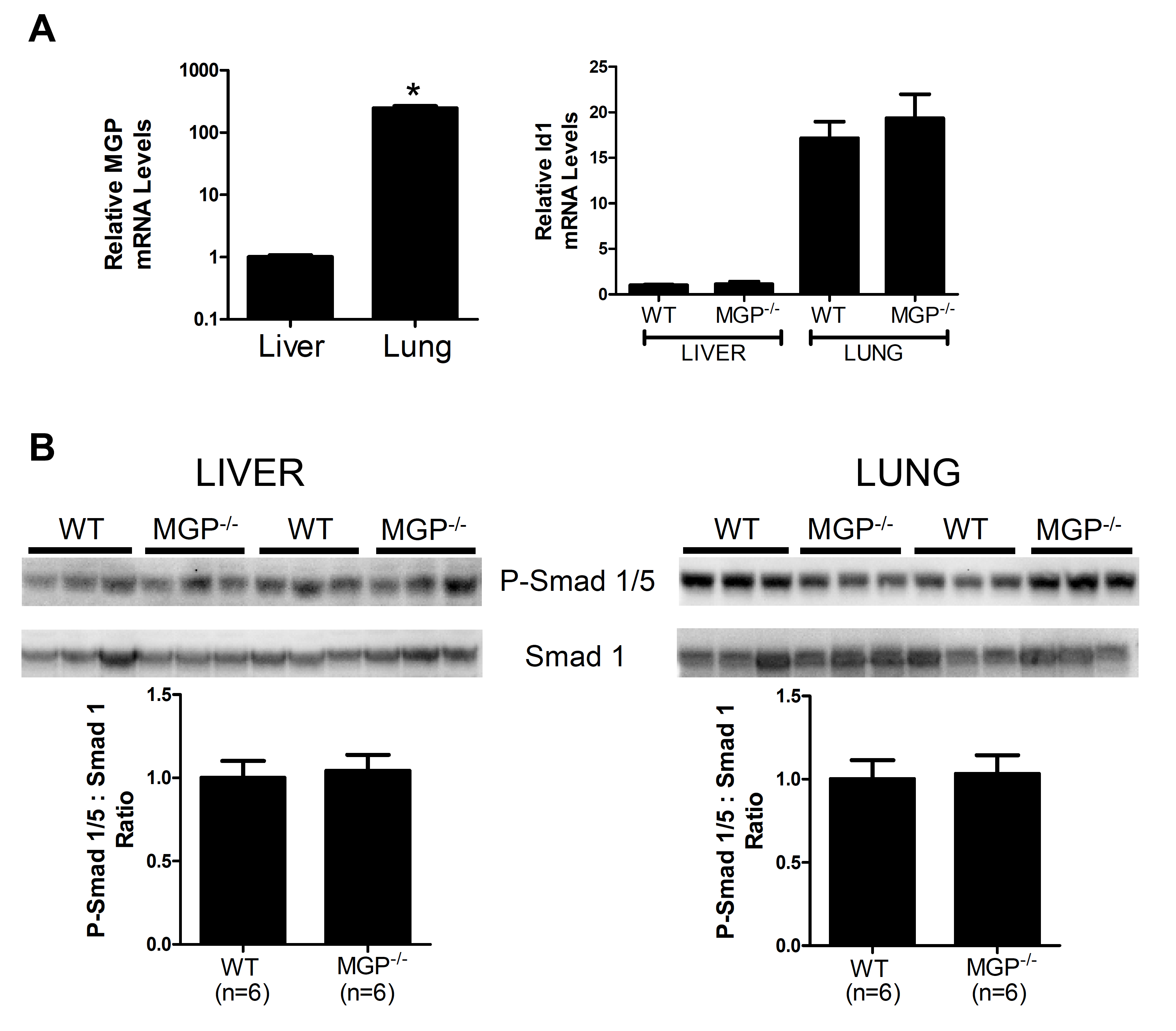

Supplement: S2 Fig — (A) MGP mRNA levels were more than 100-fold greater in the lungs compared with the livers of wild-type mice (left panel). *P<0.001 compared to liver MGP mRNA levels. No difference in Id1 mRNA levels was detected between WT and MGP-/- mice, both in the livers and lungs (n = 6 in each group, right panel). (B) Smad 1/5 phosphorylation (P-Smad 1/5) and total Smad 1 levels were measured in the livers and lungs of wild-type (n = 6) and MGP-/- mice (n = 6) at 14 days of age. There was no difference in the ratio of P-Smad 1/5 to total Smad 1 protein levels in WT and MGP-/- mice, in either liver or lung. (TIF) [file pone.0117098.s003.tif]

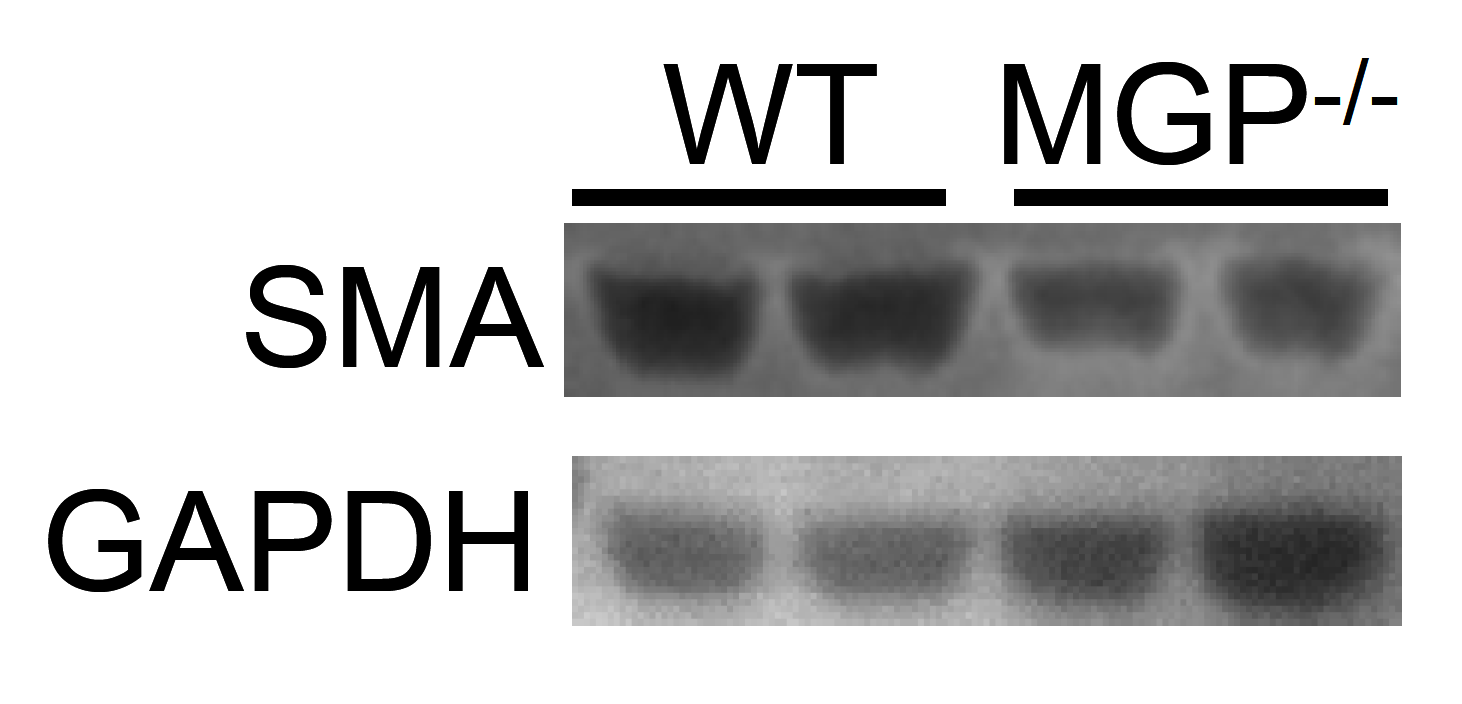

Supplement: S3 Fig — Protein lysates were harvested from aortas of WT and MGP-/- mice. PVDF membranes were treated with antibodies directed against SMA and GAPDH. Aortas from MGP-/- mice have reduced SMA protein levels compared to those of WT mice. (TIF) [file pone.0117098.s004.tif]

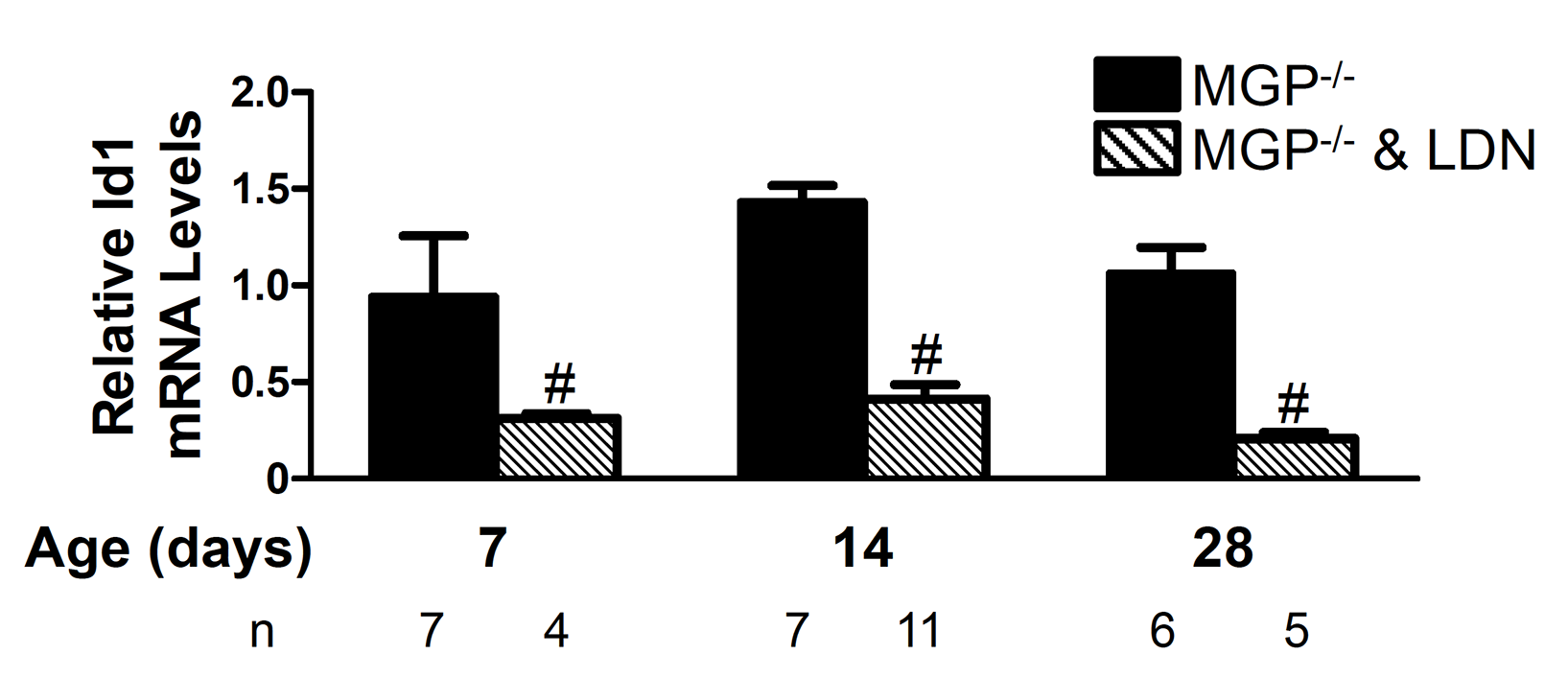

Supplement: S4 Fig — RNA was isolated from aortas of MGP-/- mice treated with either LDN-193189 or vehicle at 7, 14, and 28 days of age (n = 4–11 in each group, as indicated). Treatment of MGP-/- mice with LDN-193189 reduced aortic Id1 mRNA levels by 70–80%. # P<0.05 compared to age-matched MGP-/- mice treated with vehicle. (TIF) [file pone.0117098.s005.tif]
